# Low Myostatin Serum Levels Are Associated with Poor Outcome in Critically Ill Patients

**DOI:** 10.3390/diagnostics10080574

**Published:** 2020-08-08

**Authors:** Theresa H. Wirtz, Sven H. Loosen, Lukas Buendgens, Berkan Kurt, Samira Abu Jhaisha, Philipp Hohlstein, Jonathan F. Brozat, Ralf Weiskirchen, Tom Luedde, Frank Tacke, Christian Trautwein, Christoph Roderburg, Alexander Koch

**Affiliations:** 1 Department of Medicine III, University Hospital RWTH Aachen, Pauwelsstraße 30, 52074 Aachen, Germany; thwirtz@ukaachen.de (T.H.W.); sloosen@ukaachen.de (S.H.L.); lbuendgens@ukaachen.de (L.B.); berkan.kurt@rwth-aachen.de (B.K.); sabujhaisha@ukaachen.de (S.A.J.); phohlstein@ukaachen.de (P.H.); jbrozat@ukaachen.de (J.F.B.); ctrautwein@ukaachen.de (C.T.); 2 Clinic for Gastroenterology, Hepatology and Infectious Diseases, University Hospital Düsseldorf, Medical Faculty of Heinrich Heine University Düsseldorf, Moorenstraße 5, 40225 Düsseldorf, Germany; tom.luedde@med.uni-duesseldorf.de; 3 Institute of Molecular Pathobiochemistry, Experimental Gene Therapy and Clinical Chemistry, University Hospital RWTH Aachen, Pauwelsstraße 30, 52074 Aachen, Germany; rweiskirchen@ukaachen.de; 4 Department of Hepatology and Gastroenterology, Charité University Medicine Berlin, Augustenburger Platz 1, 13353 Berlin, Germany; frank.tacke@charite.de (F.T.); christoph.roderburg@charite.de (C.R.)

**Keywords:** intensive care unit, biomarker, sepsis, prognosis, organ failure

## Abstract

**Background:** Growth differentiation factor 8, GDF-8 (Myostatin), is a protein released by myocytes inhibiting muscle growth and differentiation. Serum concentrations of Myostatin can predict poor survival in different chronic diseases, but its role in critical illness and sepsis is obscure. Our aim was to investigate Myostatin levels as a potential prognostic biomarker in critically ill patients with sepsis. **Methods:** We therefore measured Myostatin serum concentrations in 165 critically ill patients (106 with sepsis, 59 without sepsis) upon admission to the medical intensive care unit (ICU), in comparison to 14 healthy controls. **Results:** Myostatin levels were significantly decreased in ICU patients compared to controls but did not differ in patients with or without sepsis. However, Myostatin concentrations were significantly lower in patients requiring mechanical ventilation and indicated a trend towards dependency of intravenous vasopressors. Interestingly, we observed a negative correlation between Myostatin levels and markers of systemic inflammation. Strikingly, overall survival (OS) was significantly impaired in patients with low Myostatin levels in all critically ill patients. Low Myostatin levels at baseline turned out as an independent prognostic marker for OS in multivariate Cox-regression analysis (HR: 0.433, 95% CI: 0.211–0.889, *p* = 0.023). **Conclusions:** In summary, serum Myostatin concentrations are significantly decreased in critically ill patients and associated with disease severity. Low Myostatin levels also identify a subgroup of ICU patients that are more likely to face an unfavorable clinical outcome in terms of OS.

## 1. Introduction

Growth differentiation factor 8, GDF-8 (Myostatin), represents a secreted form of the growth differentiation factor that is a member of the transforming growth factor-β (TGF-β) family. Myostatin is predominantly expressed within skeletal muscles. It is produced and released by myocytes and was shown to activate different molecular pathways resulting in inhibition of muscle differentiation, decreased protein synthesis and stimulation of protein degradation [1]. By these actions, Myostatin negatively regulates skeletal muscle growth. Results from rodent models lacking Myostatin expression or treated with inhibitors of Myostatin-activity revealed a significantly elevated muscle mass in these animals [2]. Strikingly, in mice and humans, specific mutations within the Myostatin gene are associated with a significantly enhanced muscle mass and strength [3,4]. Its clinical significance has been characterized in chronic kidney disease, where the upregulation of Myostatin in skeletal muscle was identified as one major pathway responsible for muscle wasting in patients with advanced chronic kidney disease [5,6]. These studies have raised the hope that inhibition of Myostatin may represent a therapeutic strategy in treating muscle wasting diseases such as cachexia [7,8,9].

However, the relevance of Myostatin in daily treatment of ICU patients with regard to existing and well-established scoring systems is debatable. Cachexia is a complex multifactorial syndrome associated with underlying illness and characterized by loss of skeletal muscle and fat mass. In contrast to malnutrition, cachexia cannot be reversed simply by increasing nutritional intake [10] and has been associated with an unfavorable outcome in manifold diseases [11,12,13]. Of note, up to 80% of critically ill patients treated on a medical ICU display clinical sign of cachexia underscoring the outstanding importance of this disease in the management of ICU patients [14,15]. Considering that Myostatin was shown to play a pivotal role in muscle wasting in other diseases and the fact that cachexia is a negative prognostic factor in critical illness we hypothesized that Myostatin might play a relevant role in the course of critically ill patients with and without sepsis. Therefore, we aimed at investigating Myostatin serum levels in a cohort of critically ill patients on a medical ICU at University Hospital RWTH Aachen to answer the following questions: How is Myostatin regulated in ICU patients compared to healthy controls (primary objective)? Is there an association between Myostatin levels and the severity of organ dysfunction or the presence of sepsis (secondary objective)? Might Myostatin function as a short- or long-term prognostic biomarker in ICU patients (tertiary objective)?

## 2. Materials and Methods 

### 2.1. Study Design and Patients’ Characteristics

This retrospective, observational cohort study was performed to evaluate a potential role of circulating Myostatin in critically ill patients treated on a medical ICU. We enrolled a total of *n* = 165 patients who were admitted to the Department of Gastroenterology, Digestive Disease and Intensive Care medicine between 2006 and 2011. Inclusion criteria were: 1) age above 18 years, 2) written informed consent was obtained from the patient, her/his spouse or the appointed legal guardian, 3) available blood sample at day of ICU admission. Exclusion criteria were: 1) ICU patients with an expected short-term ICU stay (<72h) e.g., due to post-operative observation, 2) patients admitted from another ICU, 3) patients admitted due to poisoning. Healthy blood donors (*n* = 14) without acute or chronic disease who are medically examined on a regular basis were included as a control population (median age: 29.5 years (18–50 years), median BMI: 25.66 kg/m^2^ (19.3–36.7 kg/m^2^), male/female: *n* = 8/*n* = 6). The Third International Consensus Definition for sepsis was used to retrospectively discriminate sepsis and non-sepsis patients [16]. The study protocol was approved by the local ethics committee (ethics committee of the University Hospital RWTH Aachen, RWTH Aachen University, Aachen, Germany, reference number EK 150/06, approved in 2006) and conducted in accordance with the ethical standards laid down in the 1964 Declaration of Helsinki.

### 2.2. Myostatin Measurements

Blood samples were collected upon admission to the ICU (day 1) and at day 7 following ICU admission. After centrifugation at 4 °C for 10 min, serum aliquots of 1 mL were frozen immediately at −80 °C until use. Myostatin serum levels were measured using a commercially available ELISA according to the instructions (Myostatin EIA Kit, Immundiagnostik AG, 64625 Bensheim, Germany). Myostatin measurements were performed fully blinded to any clinical or other laboratory data of the patients or controls.

### 2.3. Statistical Analysis

Data are given as median and range due to the skewed distribution of most of the parameters. Box plot graphics are used to illustrate differences between subgroups. All values have been included for statistical analyses. Differences between two groups were assessed by Mann–Whitney U-test. Receiver operating characteristic (ROC) curve analysis was used to assess the value of a predictive marker or a composite score. ROC curves were generated by plotting sensitivity against 1—specificity. Correlations between variables were analysed using the Spearman correlation tests. Parameters correlating with Myostatin levels at admission were included in a multivariate regression analysis with Myostatin as the dependent variable to find independent (meaningful) predictors of decreased Myostatin. To investigate a prognostic value of the variables, univariate and multivariate analysis using the Cox regression model was performed. Parameters with a *p*-value of <0.200 in univariate testing were included into multivariate testing. The hazard ratio (HR) and the 95% confidence interval are shown. In order to illustrate differences in survival Kaplan Meier curves were plotted. The Log-Rank-Test was used to test the level of significance. The ideal cut-off value for the identification of patients with an impaired survival was calculated by fitting Cox proportional hazard models to the dichotomized survival status as well as survival time and defines the optimal cut-off as the point with the most significant split in the log-rank test [17]. All statistical analyses were performed with SPSS Version 23 (SPSS, Chicago, IL, USA).

## 3. Results

### 3.1. Patients’ Characteristics

A total of *n* = 165 patients who were admitted to ICU for critical illness were included into this analysis. The median age of the study cohort was 64 years with a range from 18 to 90 years. 57.6% of ICU patients were female and 42.4% were male. The underlying cause of ICU admission was distributed as follows: 64.2% sepsis, 14.5% cardiopulmonary disease, 5.5% acute pancreatitis, 4.2% decompensated liver cirrhosis, 2.4% gastrointestinal bleeding, and 1.2% acute liver failure. The main infectious focus in patients admitted due to sepsis was pulmonary (54.7%). 64.2% of patients fulfilled the criteria of sepsis. Detailed patient characteristics are summarized in Table 1.

### 3.2. Myostatin Serum Concentrations Are Decreased in Critically Ill Patients 

**Figure 1:**
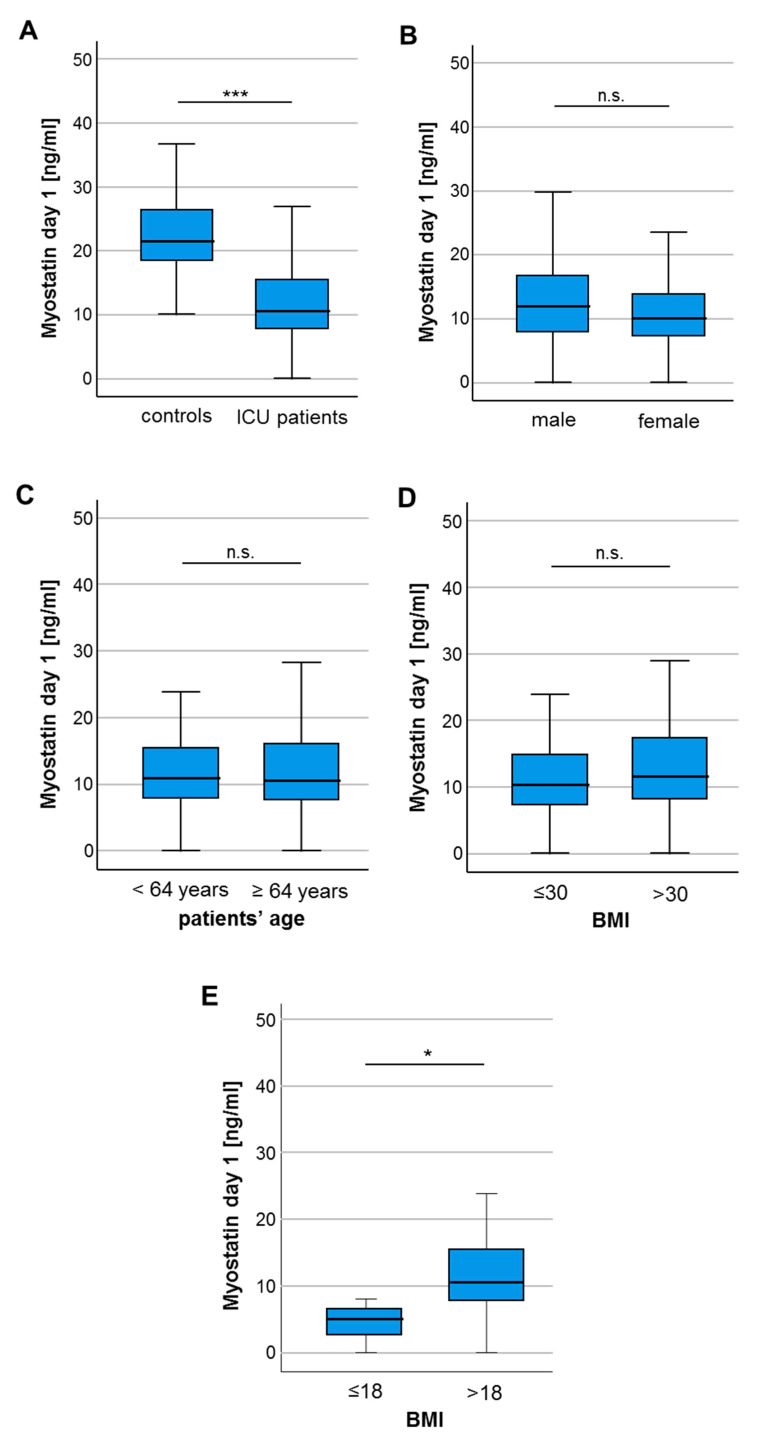
Myostatin serum levels are significantly lower in critically ill patients. (**A**) Critically ill patients at admission to the ICU have significantly lower myostatin serum levels compared to healthy controls. (**B**) Myostatin serum concentrations did neither differ between male and female patients, nor between patients younger or older than 64 years (**C**), patients with a body mass index (BMI) below or above 30 (**D**) but for patients with a BMI below or above 18 (**E**). Asterisks indicate *p* values (Mann-Whitney U-test): * *p* < 0.05, *** *p* < 0.001.

To gain a first insight into a potential regulation of circulating Myostatin in critically ill patients, we compared serum Myostatin levels between patients at the time-point of ICU admission and healthy control samples. In these analyses, we observed significantly lower Myostatin serum levels in the patients’ group (10.68 ng/mL vs. 21.61 ng/mL, *p* < 0.001; Figure 1A). Subsequently, we aimed at evaluating whether Myostatin serum levels are altered between different demographic subgroups. However, we did not observe a significant alteration of circulating Myostatin levels between male and female patients (Figure 1B), as well as patients younger or older 64 years (median of study cohort, Figure 1C). Regarding the body-mass index (BMI) of our patients, we did not find a significant difference regarding the Myostatin concentrations for patients with a BMI lower or higher than 30 kg/m^2^ (Figure 1D). Interestingly, after applying a BMI cutoff of 18 to differentiate normal from underweight patients, Myostatin concentrations differed significantly (Figure 1E).

**Figure 2:**
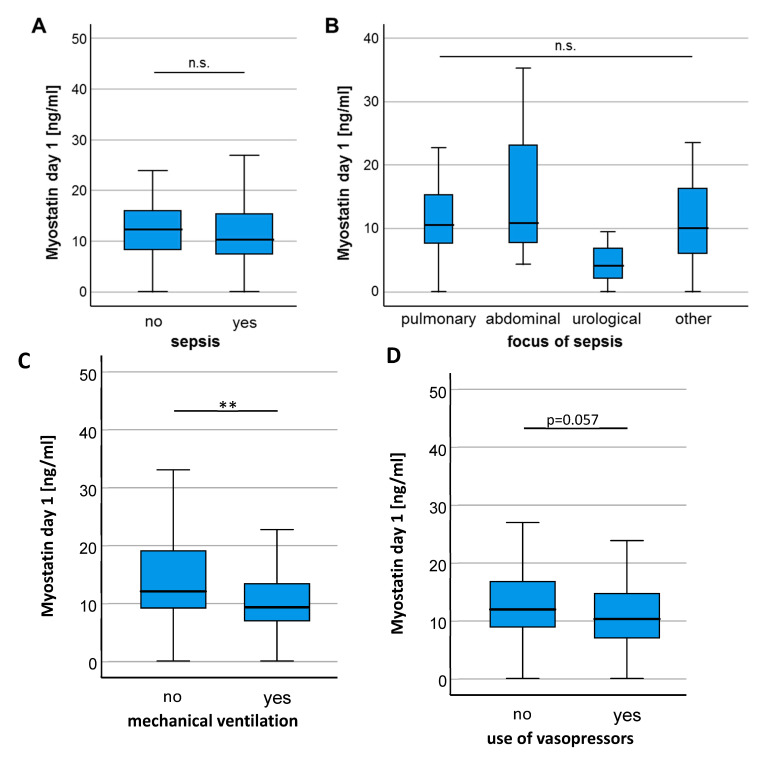

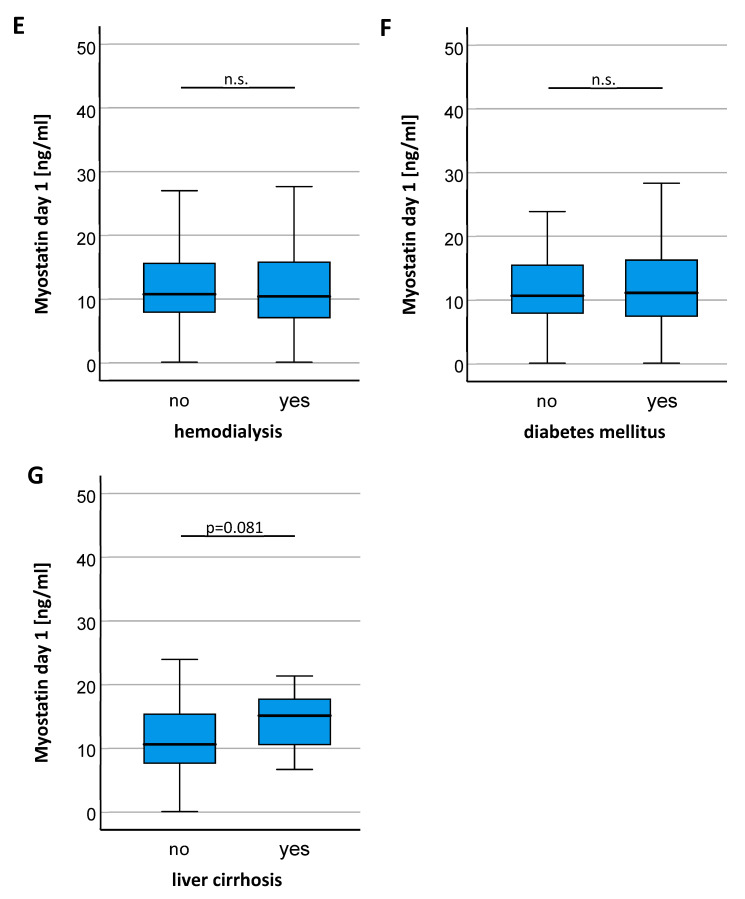
Patients requiring mechanical ventilation show a significant reduction in Myostatin levels. (**A**) Myostatin serum concentrations did not differentiate between patients admitted with or without sepsis. (**B**) Different sites of infections in patients with sepsis did not result in significantly different serum levels of Myostatin. (**C**) Patients requiring mechanical ventilation had significantly lower Myostatin levels and showed a strong trend towards use of vasopressors (**D**). No significant difference in the Myostatin level was found for indication of hemodialysis (**E**), presence of diabetes mellitus (**F**) or liver cirrhosis (**G**). Asterisks indicate *p* values (Mann-Whitney U-test): ** *p* < 0.01.

In a next step, we analyzed if individual disease characteristics of ICU patients influence circulating Myostatin levels. There was no significant difference of Myostatin serum levels between patients who did or did not fulfill the criteria for sepsis (Figure 2A) and patients with different sources of sepsis had comparable Myostatin values (Figure 2B). Interestingly, we observed significantly lower Myostatin levels in patients who required mechanical ventilation (Figure 2C) and a trend towards lower Myostatin levels in patients who depended on intravenous vasopressors at ICU admission (*p* = 0.057, Figure 2D), arguing that a more severe disease stage might be associated with lower Myostatin serum levels. In contrast, Myostatin serum levels were comparable between patients on regular hemodialysis (Figure 2E) and patients with or without diabetes mellitus (Figure 2F), while patients with liver cirrhosis showed a trend towards higher Myostatin levels compared to non-cirrhotic patients (*p* = 0.081, Figure 2G).

### 3.3. Myostatin Serum Levels in ICU Patients Negatively Correlate with Markers of Systemic Inflammation 

To further dissect potential drivers of lower Myostatin levels in ICU patients, we performed extensive correlation analyses between Myostatin serum levels and clinically established risk scores (e.g., APACHE II and SOFA) as well as a broad variety of laboratory markers indicating different types of organ dysfunction such as renal failure (creatinine), liver failure (bilirubin, AST, GGT) or systemic inflammation (leukocytes, CRP, PCT, IL-6). Interestingly, we observed a significant negative correlation between Myostatin serum levels and markers of systemic inflammation (Table 2). As such, Myostatin levels at ICU admission negatively correlated with CRP serum levels (r_S_: −0.258, *p*=0.001), PCT serum levels (r_S_: −0.240, *p* = 0.009) and IL-6 levels (r_S_: −0.342, *p* < 0.001). On the contrary, we did not observe a significant correlation between Myostatin levels and markers of an impaired liver function or kidney function (AST, ALT, bilirubin, creatinine, GFR; Table 2). Regarding further metabolic analyses, Myostatin concentrations showed a positive correlation with albumin (rs: 0.296, *p* = 0.007) but not protein serum levels (r_s_: 0.137, *p* = 0.108). Interestingly, both LDL (r_s_: 0.302, *p* = 0.023) and HDL cholesterol (r_s_: 0.310, *p* = 0.019) positively correlated with Myostatin levels, and total cholesterol levels showed a similar trend (r_s_: 0.151, *p* = 0.084). In addition to the fact that Myostatin did not differ between patients with and without sepsis, Myostatin serum levels did not correlate with clinical scores of critical ill patients as APACHE II and SOFA score (Table 2).

### 3.4. Baseline Myostatin Serum Levels Predict Overall Survival in ICU Patients

**Figure 3:**
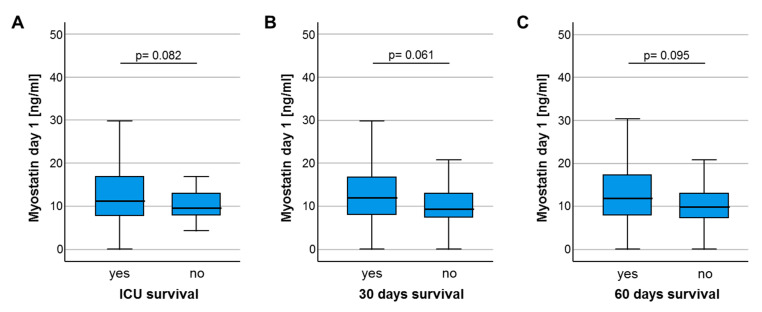

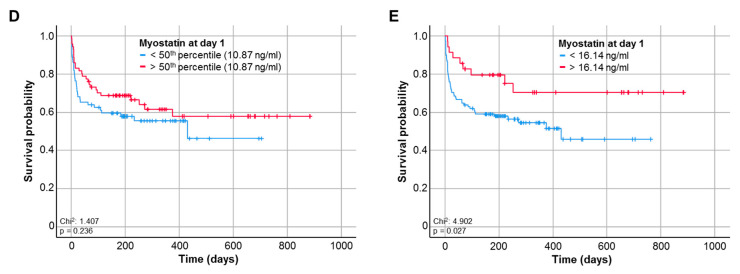
Low baseline Myostatin levels predict poor outcome in critically ill patients. (**A**) Patients who deceased on the ICU show a trend towards lower Myostatin levels compared to ICU survivors (*p* = 0.082). (**B**) Patients who deceased during the first 30 days following ICU admission show a trend towards lower serum Myostatin levels compared to survivors (*p* = 0.061). (**C**) Patients who deceased during the first 60 days following ICU admission show a trend towards lower serum Myostatin levels compared to survivors (*p* = 0.095). (**D**) Using the median Myostatin serum level (10.87 ng/ml) as cut-off value, Kaplan–Meier curve analysis reveals a trend towards an impaired overall survival (OS) in patients with low Myostatin serum levels at ICU admission. (**E**) When applying the ideal cut-off value, patients with a baseline Myostatin serum levels below 16.14 ng/ml have a significantly impaired OS compared to patient with Myostatin levels above the cut-off value (*p* = 0.027).

We next hypothesized that the downregulation of circulating Myostatin in ICU patients could indicate the individual patients’ short- and/or long-term outcome. We therefore first compared serum Myostatin levels in patients who survived the ICU stay and were discharged to a standard care ward and patients who deceased on the ICU. Here, we observed a trend towards lower Myostatin values in patients who did not survive the ICU stay, though statistical significance was not reached (*p* = 0.082, Figure 3A). In line, other time-points of short-term survival (e.g., 30 days, 60 days) revealed a similar trend towards lower Myostatin levels in patients who did not survive the respective time-point (*p* = 0.061, Figure 3B and *p* = 0.095, Figure 3C). Assuming that lower Myostatin levels might be a reflection of metabolic alterations that rather affect long-term outcome than short-term mortality, we compared the overall survival (OS) in ICU patients with high or low Myostatin serum levels. Using the median Myostatin serum level (10.87 ng/ml) as cut-off value, Kaplan–Meier curve analysis revealed a trend towards an impaired OS in patients with low Myostatin serum levels at ICU admission (*p* = 0.236, Figure 3D). We subsequently established an ideal prognostic cut-off value (see Materials and Methods for details). When applying this ideal cut-off value, patients with a baseline Myostatin serum levels below 16.14 ng/mL had a significantly impaired OS compared to patients with Myostatin levels above the cut-off value (*p* = 0.027, Figure 3E). The median OS in the “Myostatin-low group” was 430 days, but was not reached in the “Myostatin-high group”.

To further underline the prognostic value of circulating Myostatin and to exclude potential confounders, we next performed uni- and multivariate Cox-regression analysis. In univariate Cox-regression analysis below the ideal cut-off value the Myostatin serum concentration turned out as a significant prognostic factor for OS (HR: 0.458, 95% CI: 0.224–0.934, *p* = 0.032; Table 3). We next evaluated a wide range of clinicopathological parameters (age, sex, BMI) as well as various laboratory parameters of organ dysfunction including markers of systemic inflammation (leukocyte count, CRP, PCT, LDH) and an impaired liver (bilirubin, AST, ALT), renal (creatinine) or bone marrow function (hemoglobin, platelets) in univariate Cox-regression analysis. In multivariate Cox-regression analysis including parameters with a potential prognostic relevance in univariate testing (*p* < 0.200), baseline Myostatin serum levels below the ideal cut-off value turned out as an independent prognostic marker for OS (HR: 0.433, 95% CI: 0.211–0.889, *p* = 0.023; Table 3).

### 3.5. Myostatin Serum Levels during the Course of Critical Illness

**Figure 4:**
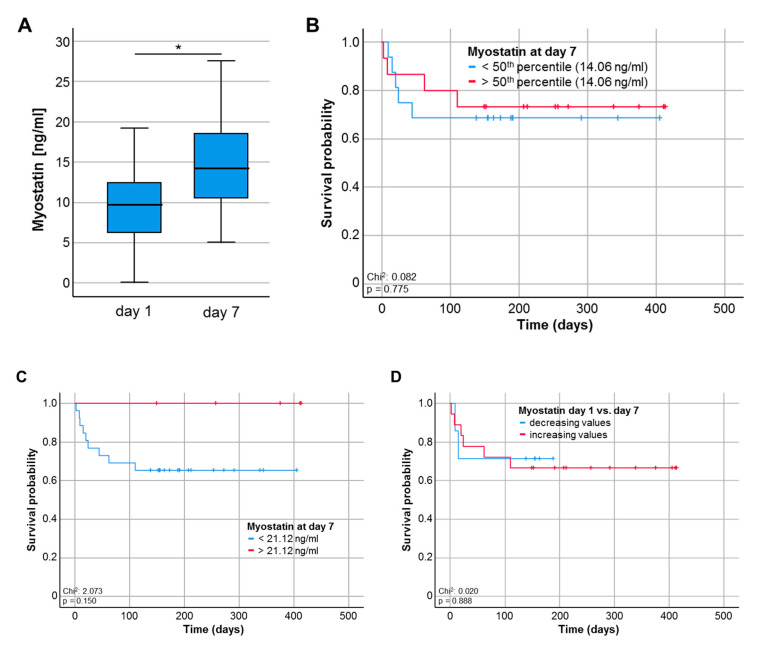
Myostatin serum levels during the course of critical illness. (**A**) Myostatin serum levels are significantly higher at day 7 following ICU admission compared to the respective levels at day 1, * *p* < 0.05. (**B**,**C**) Patients with Myostatin serum levels below the 50th percentile (14.06 ng/mL) or the ideal prognostic cut-off value (21.12 ng/ml) at day 7 following ICU admission show a trend towards an impaired OS (*p* = 0.150). (**D**) ICU patients with increasing or decreasing Myostatin levels between day 1 and 7 have a comparable OS (Figure 4D).

For a smaller subset of patients (*n* = 33), serum samples at day 7 following ICU admission were available. Interestingly, Myostatin serum levels were significantly higher at day 7 when compared to the respective values at ICU admission though the median serum level was still lower compared to healthy controls (*p* = 0.011, Figure 4A). To evaluate whether the prognostic role of circulating Myostatin was preserved during the course of ICU treatment, we again compared the overall survival between patients with high or low Myostatin values at day 7. Although statistical significance was neither reached for the 50th percentile (14.06 ng/mL, Figure 4B) nor the ideal prognostic cut-off value (21.12 ng/mL, Figure 4C), patients with low Myostatin serum levels at day 7 following ICU admission showed a trend towards an impaired OS (*p* = 0.150, Figure 4C). Finally, we analysed if the individual course of Myostatin serum levels might have an impact on the patients’ OS. However, patients with increasing or decreasing Myostatin levels (between admission and day 7) had a comparable OS (Figure 4D).

## 4. Discussion

We show that serum levels of Myostatin are decreased in critically ill patients at the time point of admission to the ICU, when compared to healthy controls. Low Myostatin levels were indicative for an unfavorable clinical outcome since patients with low Myostatin concentrations had a more complicated clinical course and displayed an impaired overall survival.

Our results from a large and well characterized cohort of critically ill patients complement previous data demonstrating a significant drop in Myostatin levels at day 4 and 30 after orthopedic or cardiovascular surgery [18]. To investigate potential drivers of low Myostatin levels in critically ill patients, Myostatin levels correlation analyses with markers of systemic inflammation and infection were performed. Here, Myostatin correlated with serum levels of C-reactive protein and Procalcitonin, reflecting a systemic inflammatory response. The assumption that Myostatin serum concentrations are linked to systemic inflammation is supported by previous animal studies: Rats that underwent cecal ligation and puncture for induction of sepsis revealed reduced *myostatin* mRNA levels in muscle tissue from septic rats in contrast to muscle tissue from control animals [19]. Interestingly, in our cohort of patients, Myostatin concentrations were not further decreased in patients that fulfilled sepsis criteria (compared to patients with systemic inflammatory distress syndrome, SIRS) highlighting that rather the presence of systemic inflammation than bacterial infection is the main driver of decreased Myostatin serum concentrations in critically ill patients. Even if Myostatin was not able to indicate presence of sepsis, it correlated with deterioration of organ function since patients depending on mechanical ventilation and intravenous vasopressors showed lower Myostatin levels. In summary, these data highlight the complex pathophysiology of Myostatin serum levels in the context organ dysfunction and inflammation and should trigger further molecular research, e.g., using myostatin^−/−^ mice to unravel the role of Myostatin as a marker in the context of critical illness and systemic inflammation.

Our cohort of critically ill patients included 24 patients with decompensated liver cirrhosis as underlying disease leading to the admission to the ICU. Interestingly, patients with liver cirrhosis displayed a trend (*p* = 0.081) towards higher Myostatin serum concentrations in comparison to patients with other etiology of critical illness. These data fit to previous studies on patients with chronic liver disease that not only revealed increased serum levels of Myostatin in patients with liver cirrhosis but also its association with worse survival [20,21]. Both liver cirrhosis as well as chronic inflammation represent diseases that are often accompanied by muscle wasting and sarcopenia. Even if sarcopenia is a common characteristic of elderly and moribund patients, its pathogenesis remains obscure. What we know so far is that muscle wasting can be triggered by manifold disease conditions including disuse, denervation, fasting, cancer, cardiac failure and renal dysfunction [22]. All of these factors are frequently found in critically ill patients. Since sarcopenia represents a negative prognostic factor for chronic diseases, we argue here that there is a need to diagnose sarcopenia at the earliest opportunity. However, in case of critical illness, assessing the body composition of an individual patient might be hampered by fluid overload [19] and the fact that classical functional tests for estimating body composition and physical strength (body weight, waist circumference, BMI, ability to walk or physical activity, and handgrip) might not provide reliable results [19]. Therefore, serum-based markers represent a desirable diagnostic tool to allow early diagnosis of sarcopenia.

Regarding a general approach to a patient’s nutrition status we first applied the BMI to investigate differences in Myostatin concentrations in patients with and without obesity. Even if Myostatin did not indicate obese patients with a BMI > 30kg/m^2^ it was significantly decreased in underweight patients. Previous studies extensively addressed the role of Myostatin in obesity as well as caloric restriction. Muscle Myostatin expression was initially shown to decline after weight loss [22]; however, in chronic diseases accompanied by cachexia such as chronic obstructive pulmonary disease (COPD) patients with higher Myostatin levels had a lower BMI [23]. Since our study lacks further long-term information on muscle mass and body composition of our patients, we tested whether Myostatin could indicate changes in metabolic pathways in critically ill patients at least for the short ICU admission time period. Therefore, Myostatin levels were correlated with markers of muscle- as well as lipid and protein metabolism: Here, Myostatin levels positively correlated with serum concentrations of LDL and HDL. The role of Myostatin in adipogenesis and diet-induced obesity is of interest in current studies since previous studies showed a suppression of body fat accumulation in *myostatin*-deficient mice [24]; however, this effect seems to be context-dependent [25]. Regarding protein metabolism, Myostatin also correlated with albumin and showed a trend for whole protein which is in line with current data on proteolysis induced by Myostatin [26]. Concerning myocyte-specific metabolism we found a negative correlation for creatine kinase contradicting a Myostatin-dependent deterioration of muscle mass in these critically ill patients. As it is unlikely that Myostatin serum levels will be clinically implemented as a stand-alone biomarker, further studies on anabolic and catabolic pathways as well as muscle metabolism and the subsequent combination with Myostatin serum levels e.g., in terms of a prognostic score are needed. Hereby, the prognostic function of Myostatin levels especially in patients treated on a medical ICU and affected by sarcopenia could be further elucidated.

Interestingly, we observed a trend towards a normalization of Myostatin concentrations between admission and day 7 of ICU treatment, which is in line to recently published results [18]. Notably neither Myostatin levels at day 7 of ICU treatment nor the difference between admission and day 7 were predictive for the patients’ outcome, which might be explained by the very small sample size at day 7. Regarding overall survival, low Myostatin levels at admission to the ICU were indicative for an unfavorable clinical outcome. At the respective optimal cut-off value that was established using a recently described biometric software [17], low Myostatin levels (<16.14 ng/mL) turned out as powerful predictor of OS. Strikingly, our data are in line to previous results from patients with cancers, where low Myostatin levels were associated with an increased long-term mortality [27,28]. These data indicate that there might be a prognostic advantage of increased Myostatin quantities. However, a potential benefit of higher Myostatin concentrations requires further investigation since previous studies describe contradictory results, especially regarding the treatment of sarcopenia: For example, the antagonism of Myostatin enhanced muscle regeneration during sarcopenia and reduced muscular dystrophy in mice [29,30]. Based on these results, clinical trials had been started to evaluate a benefit of interventions with myostatin inhibitors [31,32]. The human monoclonal antibody bimagrumab binds to Activin receptor type 2, thereby inhibiting the interaction of the ligands Myostatin with Activin resulting in skeletal muscle hypertrophy [32,33]. Future studies should therefore address both the advantageous effects on muscle growth as well as potential side effects with relevance for long-term survival in critically and chronically ill patients.

We acknowledge some limitations of our study. First, all analyses were performed in an exploratory, single-centre cohort leading to a potential lack of generalizability of results. Second, we included critically ill patients with different underlying disease aetiology. While this approach provides a certain amount of transferability of results, it might result in disease-specific confounders. Although we were able to evaluate Myostatin serum levels in a small subset of patients at day 7 following ICU admission, our study lack Myostatin evaluation at multiple time-points during ICU treatment. We established an ideal prognostic cut-off value that best discriminates between ICU patients with a favourable or unfavourable outcome. Importantly, this cut-off value does only apply to our cohort of patients and clearly needs further validation before clinical implementation. Together, our results need to be validated in larger, multi-centre cohorts of ICU patients to fully understand the regulation of Myostatin in critically ill patients and its role in predicting patient’s outcome.

In summary, we measured serum concentrations of Myostatin in a large and well characterized cohort of critically ill patients and evaluated the potential value of this parameter for estimating patients’ outcome. Despite our analysis bears important limitations such as the retrospective single-center design and a missing validation cohort, it is the first to demonstrate a potential prognostic value of Myostatin serum levels in the context of critically ill patients. Such data might provide further information for early clinical decision making on patients in emergency departments or ICUs.

## Figures and Tables

**Table 1:** Baseline patient characteristics at the time point of admission and Myostatin serum concentrations at days 1 and 7.

Parameter	Patients
Number	165
Gender	
Female, %	57.6
Male, %	42.4
Age, median, range (years)	64 (18–90)
BMI, median, range (years)	25.9 (15.9–86.5)
Diabetes mellitus type 2, %	29.7
Coronary artery disease, %	22.8
COPD, %	31.7
Main diagnosis/reason for admission, % (N)	
Sepsis	64.2 (106)
focus of sepsis, % (N)	
Pulmonary	54.7 (58)
Abdominal	19.8 (21)
Urinary tract	2.8 (3)
Other	22.6 (24)
Liver cirrhosis	4.2 (8)
Cardiopulmonary disease	14.5 (23)
Acute liver failure	1.2 (2)
Acute pancreatitis	5.5 (9)
Gastrointestinal bleeding	2.4 (4)
Other	7.9 (13)
APACHE-II score at day 1 (points, median and range)	18 (3–48)
<18, % (N)	51.4 (75)
>18, % (N)	48.6 (71)
SOFA score at day 1 (points, median and range)	9 (0–17)
<9, % (N)	56.7 (51)
>9, % (N)	43.3 (39)
Mechanical ventilation demand at day 1, % (N)	45.5 (75)
Vasopressor demand at day 1, % (N)	62.4 (103)
Death on ICU, % (N)	18.9 (30)
30d mortality, % (N)	24.3 (37)
60d mortality, % (N)	28.5 (44)
Overall mortality, % (N)	36.4 (59)
Myostatin (ng/mL)	
Day 1	10.68 (0.1–44.46)
Week 1	14.23 (0.1–27.59)

Data are given as available in % and N (in parenthesis). For quantitative variables, median and range (in parenthesis) are given. *Abbreviations are*: BMI, body mass index; COPD, chronic obstructive pulmonary disease; SOFA, sequential organ failure assessment; APACHE, Acute Physiology and Chronic Health Evaluation; ICU, intensive care unit.

**Table 2:** Correlations of Myostatin with baseline characteristics, markers of inflammation, other laboratory markers, and clinical scores at day 1 of ICU admission.

	r	*p*
**Baseline characteristics**		
Weight	0.127	0.120
BMI	0.78	0.341
**Markers of inflammation**		
Leukocytes	0.051	0.529
CRP	−0.258	0.001 **
Procalcitonin	−0.240	0.009 *
IL-6	−0.342	<0.001 ***
**Laboratory markers**		
Sodium	−0.004	0.959
Kalium	−0.072	0.373
Magnesium	0.241	0.012 *
Calcium	0.202	0.012 *
Creatinine	0.059	0.460
Urea	0.095	0.238
GFR	−0.014	0.887
Bilirubin total	0.024	0.764
AST	−0.129	0.118
γGT	−0.145	0.072
Lipase	−0.233	0.007 *
Prothrombin time	−0.173	0.033 *
Protein	0.137	0.108
Albumin	0.296	0.007 *
CK	−0.144	0.075
Lactate	−0.014	0.866
LDH	−0.014	0.864
NT-proBNP	−0,055	0.591
Fibrinogen	−0.426	<0.001 ***
**Lipid and glucose metabolism**		
Glucose	−0.120	0.136
Insulin	0.229	0.087
C-Peptide	0.139	0.304
HbA1c	−0.095	0.469
Cholesterol	0.151	0.084
LDL	0.302	0.023 *
HDL	0.310	0.019 *
Triglycerides	0.025	0.778
**Clinical Scores**		
APACHE II	−0.046	0.594
SOFA	0.002	0.988

Spearman rank correlation test was used to test significance; the Spearman’s rho correlation coefficient is depicted as “r” with * *p* < 0.05; ** *p* < 0.005, *** *p* < 0.001. Abbreviations: BMI, body mass index; CRP, C-reactive protein; IL-6, interleukin 6; suPAR, soluble urokinase-type plasminogen activator receptor; TNF-α, tumor necrosis factor α; AST, aspartate aminotransferase; γGT, gamma-glutamyl transpeptidase; CK, creatin kinase; LDH, lactate dehydrogenase; BNP, brain natriuretic peptide; LDL, low density lipoprotein; HDL, high density lipoprotein; APACHE, acute physiology and chronic health evaluation score; SOFA, sepsis-related organ failure assessment score; SAPS2, simplified acute physiology score.

**Table 3:** Uni- and multivariate Cox-regression analysis.

Parameter	Univariate Cox Regression		Multivariate Cox Regression	
	*p*-Value	Hazard-Ratio (95% CI)	*p*-Value	Hazard-Ratio (95% CI)
Myostatin >16.14 ng/mL	0.032 *	0.458 (0.224–0.934)	0.023 *	0.433 (0.211–0.889)
Age	0.001 **	1.036 (1.015–1.05)	0.004 **	1.031 (1.010–1.053)
Sex	0.928	1.024 (0.605–1.735)		
BMI	0.283	0.982 (0.950–1.015)		
Sodium	0.344	1.020 (0.979–1.062)		
Potassium	0.790	0.950 (0.654–1.382)		
Leukocytes	0.061	1.018 (0.999–1.036)	0.486	1.008 (0.986–1.029)
Hemoglobin	0.043 *	0.988 (0.977–1.000)	0.103	0.988 (0.975–1.002)
Platelets	0.078	1.001 (1.000–1.003)	0.572	1.001 (0.999–1.002)
AST	0.261	0.999 (0.997–1.001)		
ALT	0.185	0.998 (0.995-1.001)	0.170	0.998 (0.996–1.001)
LDH	0.329	1.000 (0.999–1.000)		
Bilirubin	0.964	0.997 (0.887–1.122)		
PCT	0.658	1.002 (0.994–1.009)		
CRP	0.048 *	1.003 (1.000–1.006)	0.464	1.001 (0.998–1.004)
Creatinine	0.754	1.016 (0.919–1.124)		

Abbreviations: CI, confidence interval; BMI, body mass index; AST, aspartate aminotransferase; ALT, alanine aminotransferase; LDH, lactate dehydrogenase; PCT, procalcitonin; CRP, C-reactive protein. * *p* < 0.05, ** *p* < 0.01.

## Abbreviations

ICU	medical intensive care unit
APACHE	acute physiology and chronic health evaluation score
SOFA	sequential organ failure assessment score
SAPS2	simplified acute physiology score
BMI	body mass index
CRP	C-reactive protein
IL-6	interleukin 6
γGT	gamma-glutamyl transpeptidase
AST	aspartate aminotransferase
BNP	brain natriuretic peptide
GFR	glomerular filtration rate
LDH	lactate dehydrogenase
SIRS	systemic inflammatory distress syndrome
OS	overall survival
